# Structural Determinants of CX-4945 Derivatives as Protein Kinase CK2 Inhibitors: A Computational Study

**DOI:** 10.3390/ijms12107004

**Published:** 2011-10-20

**Authors:** Hongbo Liu, Xia Wang, Jian Wang, Jinghui Wang, Yan Li, Ling Yang, Guohui Li

**Affiliations:** 1 Chemistry and Chemical Engineering School, Northeast Petroleum University, Daqing 163000, China; E-Mails: dqliuhongbo@126.com (H.L.); mrwj@nepu.edu.cn (J.W.); 2 Center of Bioinformatics, Northwest A&F University, Yangling 712100, Shaanxi, China; E-Mail: fishery18@163.com; 3 Department of Materials Sciences and Chemical Engineering, Dalian University of Technology, Dalian 116023, Liaoning, China; E-Mail: jhwang_dlut@163.com; 4 Laboratory of Pharmaceutical Resource Discovery, Dalian Institute of Chemical Physics, Graduate School of the Chinese Academy of Sciences, Dalian 116023, Liaoning, China; E-Mail: yling@dicp.ac.cn; 5 Laboratory of Molecular Modeling and Design, Dalian Institute of Chemical Physics, Graduate School of the Chinese Academy of Sciences, Dalian 116023, Liaoning, China; E-Mail: ghli@dicp.ac.cn

**Keywords:** CK2 inhibitors, 3D-QSAR, molecular docking, molecular dynamics

## Abstract

Protein kinase CK2, also known as casein kinase-2, is involved in a broad range of physiological events including cell growth, proliferation and suppression of apoptosis which are related to human cancers. A series of compounds were identified as CK2 inhibitors and their inhibitory activities varied depending on their structures. In order to explore the structure-activity correlation of CX-4945 derivatives as inhibitors of CK2, in the present study, a set of ligand- and receptor-based 3D-QSAR models were developed employing Comparative Molecular Field Analysis (CoMFA) and Comparative Molecular Similarity Index Analysis (CoMSIA). The optimum CoMFA (*R*_cv_^2^ = 0.618, *R*_pred_^2^ = 0.892) and CoMSIA (*R*_cv_^2^ = 0.681, *R*_pred_^2^ = 0.843) models exhibited reasonable statistical characteristics for CX-4945 derivatives. The results indicated that electrostatic effects contributed the most to both CoMFA and CoMSIA models. The combination of docking analysis and molecular dynamics (MD) simulation showed that Leu45, Lys68, Glu81, Val116, Asp175 and Trp176 of CK2 which formed several direct or water-bridged H-bonds with CX-4945 are crucial for CX-4945 derivatives recognition to CK2. These results can offer useful theoretical references for designing more potent CK2 inhibitors.

## 1. Introduction

The casein kinase-2 (CK2) is a pleiotropic, highly conserved serine/threonine protein kinase ubiquitously expressed in both the cytoplasm and the nucleus of eukaryotic cells [1–3]. The protein is comprised of tetramer which contains two catalytic subunits, α and/or α′ (37–44 kD), and two regulatory β-subunits (24–28 kD) in various combinations. CK2 possesses constitutive catalytic activity with the ability to phosphorylate more than 300 physiological substrates and does not require phosphorylation by other kinases for activation [2]. Naturally, these features make CK2 appear at extremely diverse points of cell signaling pathways, including PI3K/Akt and Wnt signaling cascades, NF-κB transcription, and the DNA damage response (reviewed in refs. [1] and [4]), and be involved in a number of cellular events contributing to the development of various disorders, particularly cancer. These data, in conjunction with the observation that many viruses exploit CK2 as phosphorylating agent of proteins essential to their life cycle [2], have made CK2 an attractive yet underexploited new therapeutic target for the treatment of cancer [5].

To study the function of protein kinase CK2, an effective approach is the use of small molecule inhibitors. Thus, intensive efforts have been devoted toward the development of new potent and selective CK2 inhibitors which provides a powerful tool to extend our knowledge about CK2 function as well as to regulate its activity both in case of health and disease. Most protein kinase inhibitors of practical interest, including many of those that have entered clinical practice, are competitive with respect to the phosphodonor substrate ATP, which indicates that the binding modes of inhibitors and ATP are mutually exclusive [6]. Compared with most of other protein kinases, CK2 has a smaller ATP binding site as a result of the presence of unique bulky residues [7], allowing for the design of highly selective low molecular weight ATP-competitive inhibitors [8,9]. Up to now, many classes of ATP-competitive CK2 inhibitors such as hydroxyantraquinones, hydroxylcoumarines, flavonoids, halogenate benzimidazoles and indoloquinazoline [6] have already been discovered, some of which achieved the promising inhibition potency in enzyme assays. Especially, some compounds like 3,8-dibromo-7-hydroxy-4-methylchromen-2-one (DBC) [10] outstand themselves by their advantage that their coumarinic scaffold does not act as a DNA intercalator, a property that reduces the side effects with respect to the emodin related inhibitors having antraquinone scaffolds. Despite these encouraging advances, development of potent and selective CK2 inhibitors still remains a difficult task for scientists [11,12]. In order to facilitate the drug discovery process, *in silico* screening and library design as a productive and cost-effective technology in design of novel lead compounds should be used in combination with experimental practices [13]. Presently, three-dimensional quantitative structure–activity relationship (3D-QSAR) method, including comparative molecular field analysis (CoMFA) [14] and comparative molecular similarity analysis (CoMSIA) [15] which have been widely used in drug design, is a useful tool to rationalize the molecular structural variations with their inhibitory activities. Together with the visualized contour maps of 3D-QSAR model, docking study and molecular dynamics (MD) simulation [12], it could provide deep insight into understanding the QSAR by taking into account the structural properties of the active site of protein, and thus could more effectively direct the design of new potential inhibitors.

Recent studies suggested that, due to its diverse pharmacological properties and therapeutic applications, CX-4945 has been regarded as the most promising candidates against CK2 [15]. To improve the medicinal properties and eliminate or reduce untoward effects of these compounds, several groups have performed a series of optimization procedures on them, resulting in some compounds with good activity both in the enzymatic and cell culture assays [15,16]. CX-4945, as the only one orally administered highly selective and potent CK2 inhibitor, has entered phase I clinical trials [16]. Thus development of new potent and selective CK2 inhibitors is a task of great importance. In this study, low energy conformation with ligand-based and receptor-based alignments was employed to build 3D-QSAR models for CX-4945 derivates. The predictive abilities of the obtained models were validated statistically with a representative test set of compounds. In addition, docking analysis and molecular dynamics (MD) simulation were also performed to elucidate the probable binding modes of these inhibitors. The combined *in silico* approaches have generated several 3D-QSAR models to gain insight into the key structural factors affecting their inhibitory activity and thus aid in designing new potent CK2 inhibitors with fewer side effects.

## 2. Materials and Methods

### 2.1. Data Sets

By removing compounds with unspecified inhibitory activity or undefined stereochemistry, a total of 50 CX-4945 analogues were taken from the literature [17]. All *in vitro* biological activities (IC_50_) were converted into the corresponding pIC_50_ (−lg IC_50_) values, which were used as dependent variables in the QSAR study. The total data set of analogues was divided into training and test sets in a ratio of 4:1. The structures and corresponding pIC_50_ values of the compounds in the training and test sets are given in Table 1. As a general rule, for a reliable 3D-QSAR model, the spread of activity should cover at least three log units, and there ideally should be a minimum of 15–20 compounds in the training set [18]. The activity range of CX-4945 derivatives is from 5.900 to 9.000 pIC_50_ units (see Table 1), covering four log activity distribution intervals, and there were 40 compounds in the training set.

### 2.2. Conformational Sampling and Alignment

**Figure 1:**
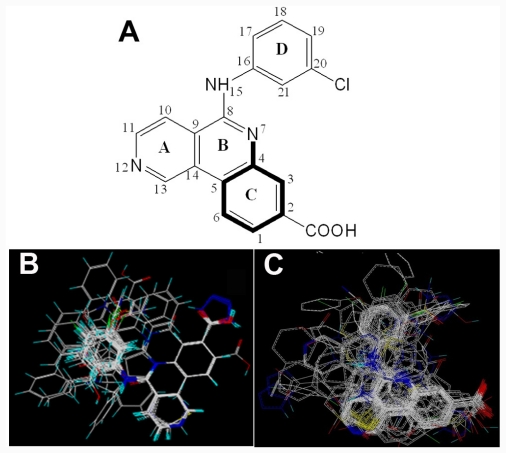
(**A**) Compound 38 used as a template for alignment. The common substructure is shown in bold. Ligand- and receptor-based alignments of all the compounds are shown in panels (**B**) and (**C**), respectively.

Molecular alignment of compounds is an important step in the development of CoMFA and CoMSIA models. To derive the best possible 3D-QSAR statistical model, two different alignment rules (ligand-based and receptor-based alignments) were adopted in this study. In the ligand-based alignment, the 3D structures of all compounds were constructed and subjected to full geometry optimization using the sketch molecule module of SYBYL 6.9 package (Tripos Associates, St. Louis, MO). Partial atomic charges were calculated by the Gasteiger-Huckel method, and energy minimization was performed by using the Tripos force field and the Powell conjugate gradient algorithm with a convergence criterion of 0.05 kcal/mol·Å. Then inhibitors were superimposed on the most potent molecule (compound 38) according to the common substructure depicted in bold (Figure 1(A)), and the resulting ligand-based alignment model is shown in Figure 1(B). In the receptor-based alignment, the protonation states of the titratable groups of CK2 were checked by using Whatif [19], the model pK_a_s for ligand titratable groups were calculated by SPARC [20]. Then computational docking was performed using Surflex module of SYBYL package. All inhibitors were aligned according to the bioactive conformations in the binding pocket of CK2α (PDB entry code: 3NGA) obtained from docking with Gasteiger Huckel charge (Figure 1(C)).

### 2.3. CoMFA and CoMSIA 3D-QSAR Models

The original setup for CoMFA and CoMSIA modeling was similar to our earlier work [13]. In summary, CoMFA was carried out using steric and electrostatic potential fields while CoMSIA was based on five fields (steric, electrostatic, hydrophobic, H-bond donors and acceptors). All the calculations were performed using Sybyl software.

In the partial least-square (PLS) regression analyses, CoMFA and CoMSIA descriptors were used as independent variables and the pIC_50_ values were used as dependent variables to derive the 3D-QSAR models. The predictive values of the obtained models were evaluated using leave-one-out (LOO) cross validation method. The correlation coefficient *R*^2^, F value and standard error of estimates (SEE) were calculated. The models were also evaluated for their ability to predict the activity of compounds in the test set. The predictive *R*^2^ (denoted by *R*_pred_^2^) was calculated by Equation 1.

(1) $${R_{\text{pred}}}^{2}=1-(\text{PRESS}-\text{SD})$$

where SD is the sum of squared deviations between the biological activities of the test set and the mean activity of the training set molecules, and PRESS is the sum of squared deviation between the actual and predicted activities of the test set molecules. The optimum number of components (ONC) corresponding to the lowest PRESS value was used for deriving the final PLS regression models.

### 2.4. Molecular Dynamics

Molecular dynamics simulations were carried out using the Amber 10 package [21] by starting the docked structure. Ligand parameters and charges were determined with the antechamber module of Amber 10 based on the general atom force field (GAFF) [22] and the AM1-BCC charge scheme [23]. The standard AMBER force field for bioorganic systems (ff03) [24] was used to describe the protein parameters. All systems were solvated in a rectangular box of TIP3P water [25], keeping a minimum distance of 10 Å between the solute and each face of the box. The system was neutralized, and 0.15 M sodium chloride was added to the simulation box. To remove possible bad contacts, the complex was energy minimized by a multistep procedure including 500 steepest-descent steps followed by 9500 conjugate-gradient steps.

Then the solvent molecules in the minimized models were heated up to 300 K and equilibrated 200 ps with the positional restraints on the protein heavy atoms. The MD simulations were performed in the NPT ensemble at constant pressure (1 atm) with isotropic position scaling and at 300 K. Finally, the production phase was run for 5 ns with a 2 fs time step. The long-range electrostatics was treated by using the particle-mesh-Ewald (PME) method [26] with default values.

## 3. Results and Discussion

Since the structural alignment of the compounds is crucial to develop a successful 3D-QSAR model, in this study, two rules (both ligand- and docking-based) are employed to align each class of compounds to derive the reliable CoMFA and CoMSIA models. The results obtained from both alignment methods using the same training set compounds are summarized in Table 2. The selected test set (representing 25% of the training set), which was not included in the model training process, was used to evaluate the predictive power of the CoMFA and CoMSIA models. There are several statistical parameters, *i.e.*, the cross-validated correlation coefficient (*r*_cv_^2^), non-cross-validated correlation coefficient (*r*_ncv_^2^), standard error of estimate (SEE) and F-statistic values (F) that are inherent in appraising a QSAR model for the compounds to be studied.

For CoMSIA models, all possible combinations of fields were evaluated to determine which of the five fields are actually needed for the generation of a predictive model, because the five descriptor fields are not totally independent of each other and such dependencies of individual fields might decrease the statistical significance of the models. In the present work all 31 possible combinations of the descriptors were attempted to build the optimum models with highest *R*_cv_^2^ values and other proper statistical results. The resultant optimum models exhibited agreeable statistical results for this class of compounds (Table 2).

### 3.1. Validation of the 3D QSAR Models

CoMFA model validated internally yielded *R*_cv_^2^ = 0.618 with three optimum components and *R*_cv_^2^ = 0.562 with four optimum components for the ligand- and docking-based alignment methods, respectively. Then the number of components identified in the LOO cross-validation process was used in the final non-cross-validated PLS run. As a consequence, the training set of 40 compounds led to *R*_ncv_^2^ = 0.860, SEE = 0.343, *F* = 71.892, *R*_pred_^2^ = 0.892 and *R*_ncv_^2^ = 0.877, SEE = 0.326, *F* = 62.523, *R*_pred_^2^ = 0.556 for ligand- and docking-based models, respectively. The electrostatic field makes more contribution to the activity (~56.7%) in ligand-based CoMFA model while less (~33.2%) in the docking-based one.

CoMSIA modeling gave *R*_cv_^2^ = 0.681 at three PLS components, *R*_ncv_^2^ = 0.828, *R*_pred_^2^ = 0.843, SEE = 0.380 and SEP = 0.518 with steric of 18.0% and electrostatic of 82.0% field contributions for the ligand-based alignment, while yielded *R*_cv_^2^ = 0.545 at five PLS components, *R*_ncv_^2^ = 0.791, *R*_pred_^2^ = 0.580, SEE = 0.187 and SEP = 0.637 with steric of 15.1%, electrostatic of 24.6%, hydrophobic of 32.1% and H-bond acceptor of 28.2% field contributions for the docking-based alignment, respectively. All these results suggested that ligand-based alignment of this class of compounds led to the models with larger *R*_cv_^2^, *R*_ncv_^2^, *R*_pred_^2^ and lower SEE, SEP values than those obtained from receptor-based alignment. Therefore, the ligand-based 3D-QSAR models of this class of compounds were chosen as the optimal model in this work and utilized for further discussion.

**Figure 2:**
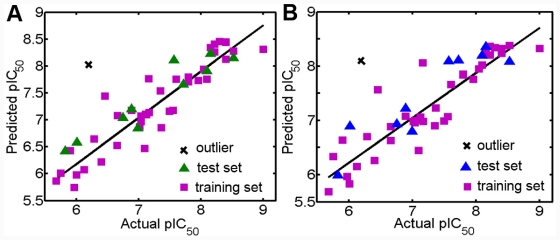
(**A**) Plot of the predicted pIC_50_ *versus* the experimental pIC_50_ values for CoMFA analysis. (**B**) Plot of predicted activities *versus* experimental activities for CoMSIA analysis. The solid lines are the regression lines for the fitted and predicted bioactivities of training and test compounds.

During the cross-validation procedure, compound 46 is detected as an outlier (whose residual between the experimental and predicted values is more than 1.5 log unit) for both CoMFA and CoMSIA models. Some reasons may cause this appearance as an outlier. Compound 46 has a unique structure feature including an amide segment probably responsible for specific interactions which may make it an outlier. To test the predictive ability of the model, a test set of ten molecules excluded from the model derivation was employed. The predictive correlation coefficients *R*_pred_^2^ of ligand-based CoMFA and CoMSIA models were 0.892 and 0.843, respectively. The average absolute residuals of the predicted *vs.* corresponding experimental pIC_50_ values were 0.256 and 0.259, respectively. The plot of actual activity *versus* predicted pIC_50_ of the training set and test set was illustrated in Figure 2. The plots represent a uniform distribution around the regression line, indicating the satisfactory predictive capability and accuracy of the model.

### 3.2. 3D-QSAR Contour Maps

CoMFA and CoMSIA contour maps are generated by interpolating the products between the 3D-QSAR coefficients and their associated standard deviations to visualize the information of the derived 3D-QSAR models. The maps depict regions having scaled coefficients greater than 80% (favored) or less than 20% (disfavored). To aid in visualization, the most active compound is shown with the contour maps which indicate regions in 3D space around the molecules where changes in the particular physicochemical properties are able to explain the experimental binding differences. The combination of CoMFA and CoMSIA approaches enables one to check the convergence of the results, or to obtain conclusions that can complement each other [27,28]. In such a case, exploiting the results of both approaches leads to an optimal interpretation at the 3D level of the QSAR [28,29]. Thus, they not only rationalize the quantitative relationship between the molecular structures and their activity, but also provide valuable structural optimization clues for drug design.

**Figure 3:**
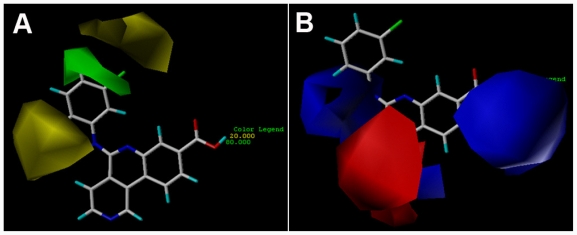
CoMFA StDev * Coeff contour plots. (**A**) Steric (green/yellow) contour map combined with compound 38. Green contours indicate regions where bulky groups increase activity; yellow contours indicate regions where bulky groups decrease activity. (**B**) Electrostatic contour map (red/blue) in combination with compound 38. Red contours indicate regions where negative charges increase activity; blue contours indicate regions where positive charges increase activity.

**Figure 4:**
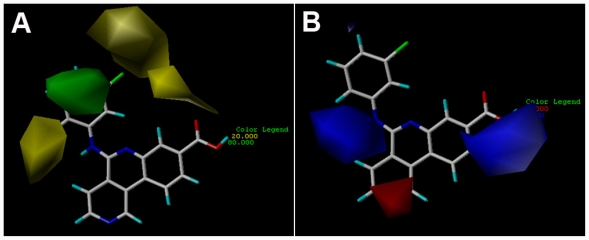
CoMSIA StDev * Coeff contour plots. (**A**) Steric (green/yellow) contour map combined with compound 38. Green contours indicate regions where bulky groups increase activity; yellow contours indicate regions where bulky groups decrease activity. (**B**) Electrostatic contour map (red/blue) in combination with compound 38. Red contours indicate regions where negative charges increase activity; blue contours indicate regions where positive charges increase activity.

The CoMFA contour maps (Figure 3A,B) generated from the model derived by steric/electrostatic field combination are the same as the CoMSIA contour maps (Figure 4A,B) obtained by steric/electrostatic field combination, indicating the convergence of the results. For steric fields, the green and yellow contours describe regions of space around the molecules, in which green colored regions indicate areas where increased steric bulk links with enhanced activity, and yellow regions suggest areas where increased steric bulk is unfavorable to activity. Compound 38 was selected as a reference molecule to aid the visualization. As illustrated in Figures 3(A) and 4(A), a median green contour is found adjacent to the positions -19 and -20 of ring D, thus molecules which carry bulkier substituents such as chlorine at position-20 (compound 38) are more active than those compounds with less bulky substitutions like fluorine at the same position (compound 36). Thereby, an addition of bulky groups at these positions around the green contour probably improves the potency of the inhibitors. Another group of CoMFA and CoMSIA sterically disfavored yellow contours are present outside ring D, which strongly delimits the size of the side-chain around ring-D. For instance, the low potency of compounds like 10, 11 and 33 is probably attributed to the presence of too bulky substituents at position-15, which conflicts with the yellow forbidden region. This suggests that the optimal length of the substituents at position-15 will enhance the activity of these compounds (32 and 38). Furthermore, the yellow contour around position-19 and -20 of ring D is farther than the green one, indicating too bulky groups at these positions are unfavorable.

In the electrostatic field contour maps of CoMSIA and CoMFA, the red contours show favorable electronegative regions, and the blue contours show the regions where the electropositive charges are favored for enhancing the bioactivity. As shown in Figures 3B and 4B, a huge blue contour around ring C indicates the importance of electropositive substituent at this position to the inhibitory activity. For examples, compound 17 with a –COOH at position-2 of ring C exhibited better potency than compound 22 which possesses a -C-(1*H*-tetrazol-5-yl) group at the same position. In addition, another electropositive favorable blue contour near position-15 suggests that maybe substituents more positively charged (like the –NH– of compound 10) than –NMe– (of compound 09) in this region are good for increasing the activity. Therefore, reasonable structural modifications can be carried out to improve the activity and selectivity of CK2α inhibitors. Another red contour near position-12 of ring A indicates that electronegative groups at this region will enhance the activity. For examples, compound 50, having relatively electropositive group (=N– at position-12) is more active than compound 49 wherein =CH– group is attached. Compound 25 having relatively electronegative group ( 
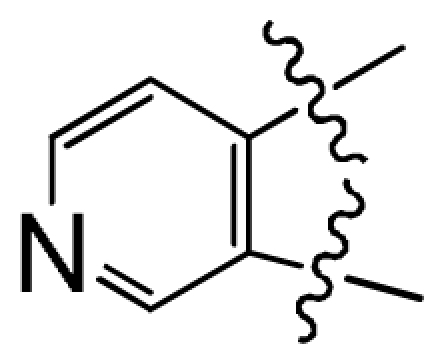
) is more active than compound 24 (with 
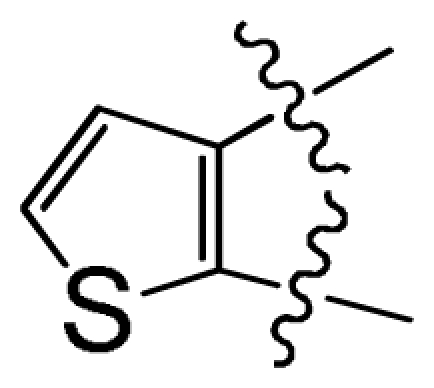
 group at the same location).

### 3.3. Docking Analysis and Comparison with 3D Contour Maps

Besides the ligand-based 3D-QSAR studies, molecular docking for all 50 inhibitors was performed not only to understand the nature of interactions between these compounds and CK2α kinase but also to complement the 3D-QSAR analysis for the rational design of drugs.

Before docking analysis, a redocking process of the cocrystallized ligand compound 38 (CX-4945) into CK2α receptor (PDB code: 3NGA) was carried out to validate the docking protocol and parameters used. The top-ranked docked solution was found in one favorable cluster of docking poses with average root-mean-square deviation (RMSD) values ranging from 0.3 to 1.9 Å for the 10 top-ranked docking poses, suggesting the binding mode is successfully reproduced. Additionally, several key residues including Lys68, Glu81, Val116, His160, and Asp175 appear in the binding cavity, confirming the reasonability of docking protocol. For this class of compounds, the docking results in the absence of the crystallized waters are poorer than those in the presence of crystallized waters. This is probably that crystallized waters are crucial for mediating the interactions between ligand and the protein.

**Figure 5:**
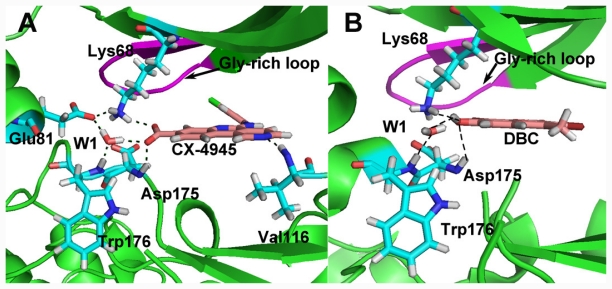
Plot of the CK2α structures of the binding site with ligand. (**A**) The binding modes between CX-4945 and CK2α (PDB code: 3NGA). (**B**) The binding modes between DBC and CK2α (PDB code: 2QC6). H-bonds are denoted as dotted black lines.

Compound 38 was selected as a reference to analyze the binding mode of docking shown in Figure 5. Compound 38 locates at a cavity consisting of Leu45, Gly46, Arg47, Val53, Val66, Lys68, Ile95, Phe113, Glu114, His115, Val116, Asn118, His160, Met163, Ile174, Asp175, and Trp176. The ligand is anchored in the binding site mainly via three H-bonds and two water-mediated contacts with the protein. The carboxyl group at position-2 of ring C forms an H-bond with side chain of Lys68 (–O···HN, 1.9 Å) and the backbone of Asp175 (–O···NH, 1.9 Å), and the nitrogen atom at position-12 of ring A forms an H-bond with the backbone of Val116 (–N···NH, 1.7 Å). The hydroxyl group at position-2 forms two H-bonds with water1 (W1), which itself forms two H-bonds to the backbone –NH– of Trp176 and side chain Glu81, respectively. Carbonyl group forms an H-bond with –OH of Asp175 through water2 (W2). These results are consistent with the CoMFA and CoMSIA electrostatic blue contour at position-2 of ring C which suggests that electropositive substituents in this area can increase the activity, and the red contour at position-12 of ring A which indicates that electronegative substituents in this area can increase the activity. Furthermore, no amino acid residues appear upon the plane of the benzene ring (ring D), indicating that bulky substituents in this position are favored for inhibitory activity. This is also evidenced by the presence of a sterically favorable green contour around this area as seen by the CoMFA and CoMSIA models. While the area above or below ring D is occupied by residues of Leu45, Gly46 and His160, suggesting that the substituents bearing side chains in this position will conflict with these residues and decrease the inhibitor activity. Therefore, the ring D substituent of compound 38 that fits snugly into this hydrophobic cleft would be preferential.

### 3.4. Comparison with Binding Modes of 3,8-Dibromo-7-hydroxy-4-methylchromen-2-one (DBC)

The binding modes of this type of inhibitors were compared with those of DBC on purpose to explore their similarities and differences and to get a better understanding of the variations in their biological activities. DBC is the derivative of coumarin which belongs to natural benzopyrone derivatives. Since benzopyrones widely exist in vegetables, fruit, seeds, nuts, coffee, tea and wine [30], it is not difficult to see why extensive study on their pharmacological and therapeutic properties has been underway over many years [30,31]. Especially, coumarin is a natural substance that has shown antitumor activity *in vivo*, with the effect believed to be due to its metabolites (e.g., 7-hydroxycoumarin) [31].

Based on the docking study, we found that H-bond and water-mediated interactions are both important between the CX-4945 inhibitors and the CK2α receptor. For CX-4945 (shown in Figure 5A), three direct H-bonds are formed between compound 38 and residues Lys68, Val116 and Asp175. Water molecule (W1) mediated interactions are formed between compound 38 and residues Glu81 and Trp176. As regards DBC (shown in Figure 5B), it locates at a hydrophobic cavity consisting of the side chains of Leu45, Gly46, Val53, Lys68, Ile66, Phe113, Glu114, Val116, Met163, Ile174, Asp175, Trp176 and Gly177. DBC hydroxyl group forms a direct H-bond with residues Lys68 (–O···HN, 1.8 Å) and Asp175 (–O···HN, 2.5 Å), respectively. Furthermore, the hydroxyl group of DBC establishes another H-bond with Trp176 backbone via a water molecule (W1), which further confirms that this structure is crucial to the DBC inhibitory activities.

By comparison, we obtained the following conclusions: (1) Note that the DBC is always co-planar, but in the CX-4945 case the inhibitor is displaced laterally so that it overlaps different sections of the hydrophobic cavity, and in the latter it enters the cavity deeper than the DBC, reaching the hinge region, where it establishes more bonds with the receptor; (2) Lys68 and Asp175 are both involved in the binding modes, indicating that both of them are important for the interaction between these two series of inhibitors and the CK2α protein; (3) Both inhibitors form more than two H-bonds with the CK2α, indicating that they exhibit potent inhibitory activity; (4) Besides the direct H-bond, the interaction mediated by water is also vital for the recognition process of both classes of inhibitors. Particularly, water molecule that mediates an H-bond between the OH group of inhibitors and the Trp176 backbone of CK2α may be conserved.

### 3.5. MD Simulations Analysis

**Figure 6:**
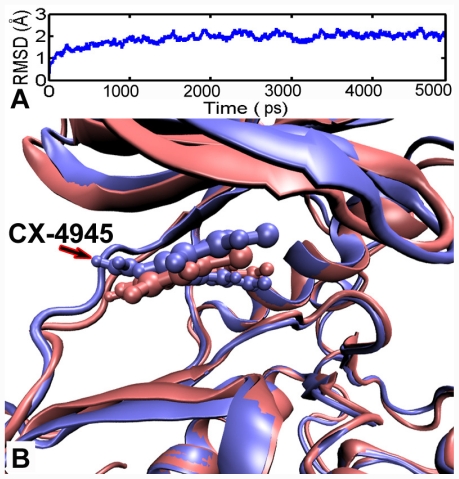
(**A**) Time evolution of the root-mean-square deviation (RMSD) of CK2α measured from the corresponding initial structure. (**B**) View of superimposed backbone atoms of the average structure of the molecular dynamics (MD) simulation (magenta) and the starting structure (blue) for CK2α. CX-4945 is shown as ball and stick in magenta for the average structure and in blue for initial complex, respectively.

To investigate the positional and conformational changes of inhibitors relative to the binding site, a 5 ns MD simulation was performed based on the crystal structure of CK2α in complex with CX-4945 (PDB code:3NGA). Initially, to determine the conformational stability of the CK2α structure, the RMSD over the lifetime with respect to its starting structure, is examined. The RMSD of all backbone atoms as a function of time is depicted in Figure 6(A). After 1.5 ns, the RMSD of the complex reaches about 2 Å and retains this value throughout the simulation, indicating that the overall structure of the CK2α has reached a stable conformation in time during the simulations.

**Figure 7:**
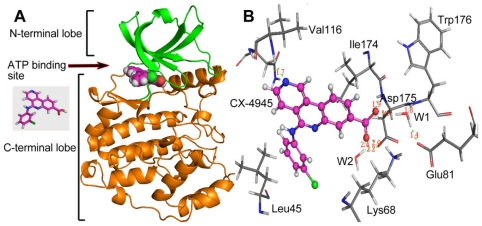
(**A**) Overall structure of the CK2α in complex with CX-4945. The *N*-terminal lobe, which is rich in β-strands and ends at Asn118, is implicated in nucleotide binding. The *C*-terminal lobe is mainly α-helical and serves as a docking site for substrates. CX-4945 binds to the ATP-binding site near the hinge region, which unites both lobes. (**B**) The binding mode between CX-4945 and CK2α. CX-4945 binds to CK2α directly or through the water molecules W1 and W2. H-bonds are shown as dotted green lines; Active site amino acid residues are represented as sticks; the inhibitor is denoted as stick and ball model.

**Figure 8:**
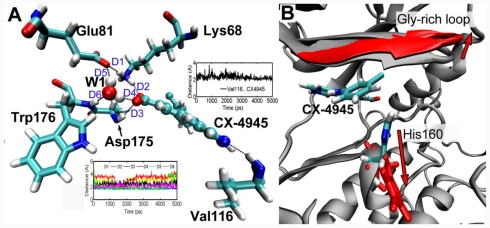
(**A**) Plot of the MD-simulated structure of the binding site with CX-4945. H-bonds are shown as dotted black lines, active site amino acid residues are represented as sticks, the inhibitor is shown as stick and ball model, W1 is represented as Vdw, respectively. Three direct (D1, D2 and D3) and W1-mediated (D4, D5 and D6) H-bonds keep stability during the whole simulation (bottom left), the direct H-bond between the nitrogen of ring A and Val116 observed from the crystal structure is also stable in the simulation (top right). (**B**) Superposition of the initial and final snapshots of the CK2α simulation. For clarity, only the Gly-rich loop is colored in gray (initial) and red (final). Residue His160 in final structure is shown in red stick presentation, while in initial structure is shown in stick presentations: cyan for carbon, white for hydrogen, red for oxygen, and blue for nitrogen atoms, respectively. As indicated by the red arrows, the Gly-rich loop moves upward while His160 flips downward, thus opening the mouth of the cleft.

For CK2α, CX-4945 is captured in the ATP binding site sandwiched between the *C*- and *N*-terminal lobes (Figure 7(A)). We found that the condensed planar structure of CX-4945 comprising three flat rings A, B and C is remarkably stable, displaying backbone RMSF values around 0.6 Å. Whereas the side chain (ring D) shows pronounced flexibility with RMSF 2.0 Å. In the CK2α binding cleft, the condensed planar structure of CX-4945 binds to the CK2α through the van der Waals contacts and hydrophobic interactions with a hydrophobic surface of the CK2α binding cleft formed by residues Leu45, Gly46, Val53, Val66, Ile95, Phe113, Met163, Ile174, Asp175 and Trp176 (Figure 8(A)). Simultaneously, three direct (between the carboxyl group of ring C and Lys68, between the carboxyl group of ring C and Asp175, and between the nitrogen of ring A and Val116) and W1-mediated H-bonds observed from the crystal structure keep stability during the whole simulation (Figure 8(A), bottom left and top right), while the W2-mediated H-bond is not conserved in the simulation. These interactions including W1-mediated H-bonds enable the CK2α to grasp the ligand tightly.

At the “mouth” of the CK2α binding cleft where side-chain ring D lies (Figure 8(B)), Gly-rich loop backbone (residues 46–51 in CK2α) as the upper lip shows a significant flexibility, which moves “up” and is shifted away from the lower lip His160. Meanwhile, the imidazolyl of His160 flips downward, thus opening the mouth of the cleft. This enlarged space of the mouth enables ring D to rotate freely to explore an optimum pose. This observation highlights the flexibility of the Gly-rich loop and His160, which is able to adjust them to the type of ligand present in the cavity.

## 4. Conclusion

In this study, the ligand-based and receptor-based 3D-QSAR studies using CoMFA and CoMSIA approaches have been performed on CX-4945 derivatives as CK2 inhibitors. From the resultant model, the established ligand-based 3D-QSAR models show good correlative and predictive ability in terms of higher Q^2^, *R*_ncv_^2^, and *R*_pred_^2^ values. The resulting contour maps produced by the best CoMFA and CoMSIA models provide useful information about the intermolecular interactions of inhibitors with the surrounding environment. The results are in good correlation with the specific interactions between the inhibitors and the binding pocket of human CK2α identified in the docking analysis. MD simulation results of CK2α in complex with CX-4945 reveal that CX-4945 forms several direct or water-bridged H-bonds with the participation of W1, Leu45, Lys68, Glu81, Val116 and Trp176. These hydrogen bonds allow CX-4945 to bind to CK2α strongly and selectively. All these results will be extremely beneficial for guiding future structural modifications and developing novel and potent CK2 inhibitors.

## Figures and Tables

**Table 1:** The structures of the training and test set molecules of CX-4945 CK2 inhibitors.

No.	Structure						IC_50_ (μM)	pIC_50_
1	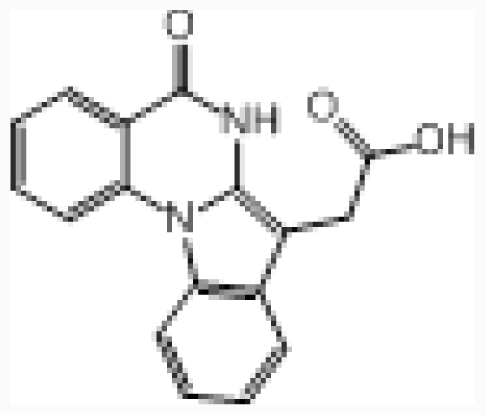						0.08	7.097

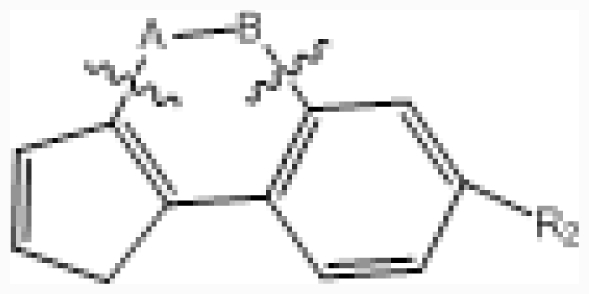								

**No.**	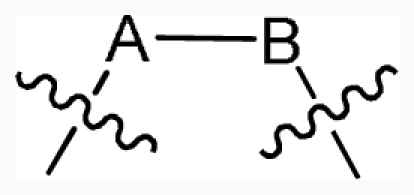			**R_2_**			**IC_50_(μM)**	**pIC_50_**

2	–CO–NH–			–COOH			2.1	5.678
3 †	–CO–N((CH_2_)_3_OH)–			–COOH			1.5	5.824
4	–(C–O(CH_2_)_3_OH)=N–			–COOH			0.99	6.004
5	–(C–NH(CH_2_)_3_OH)=N–			–COOH			0.75	6.125

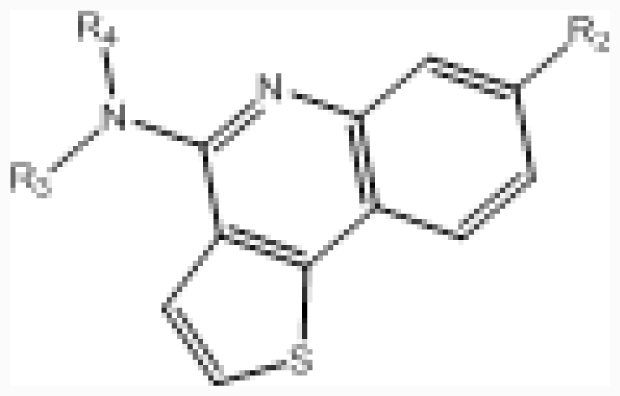								

**No.**	**–NR_3_R_4_**			**R_2_**			**IC_50_(μM)**	**pIC_50_**

6	–NH–(CH_2_)_2_OH			–COOH			1.26	5.900
7 †	–NH–(CH_2_)_2_NMe_2_			–COOH			0.102	6.991
8	–Pyrrolidino			–COOH			1.780	5.750
9	–NH–phenyl			–COOH			0.092	7.036
10	–NMe–phenyl			–COOH			1.070	5.971
11 †	–NH–(2–Me–phenyl)			–COOH			0.970	6.013
12	–NH–phenyl			–C-(1*H*-tetrazol-5-yl)			0.096	7.018
13	–NH–(CH_2_)_2_Ph			–COOH			0.516	6.287
14	–NH–(4–F–phenyl)			–COOH			0.219	6.660
15	–NH–(3–F–phenyl)			–COOH			0.068	7.168
16 †	–NH–(4–Cl–phenyl)			–COOH			0.178	6.750
17	–NH–(3–Cl–phenyl)			–COOH			0.032	7.495
18	–NH–(3–MeO–phenyl)			–COOH			0.077	7.114
19	–NH–(3–acetylenyl–phenyl)			–COOH			0.028	7.553
20	–NH–(3–(PhO)–phenyl)			–COOH			0.395	6.403
21 †	–NH–(3–(CONHMe)–phenyl)			–COOH			0.129	6.889
22	–NH–(3–Cl–phenyl)			–C-(1*H*-tetrazol-5-yl)			0.129	6.889
23	–NH–(3–F–phenyl)			–C-(1*H*-tetrazol-5-yl)			0.075	7.125

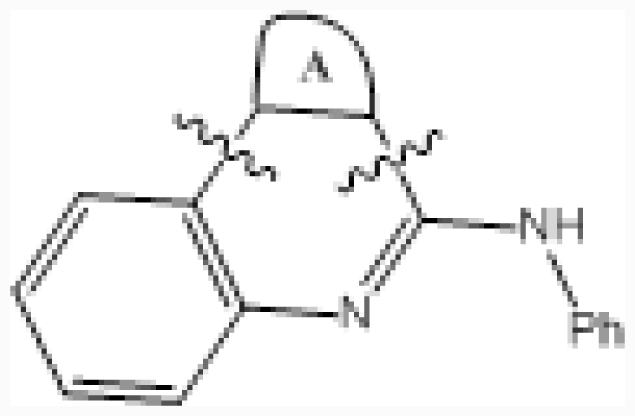								

**No.**	**A ring**						**IC_50_(μM)**	**pIC_50_**

24	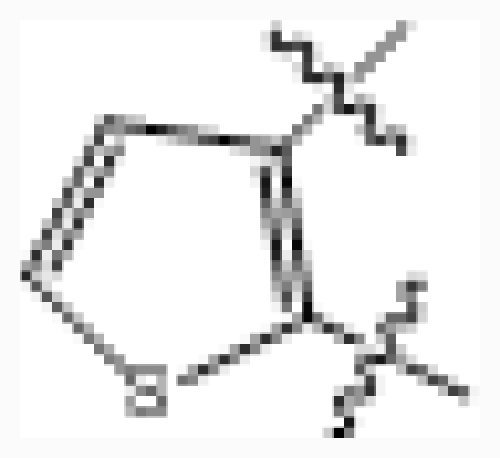						0.092	7.036
25	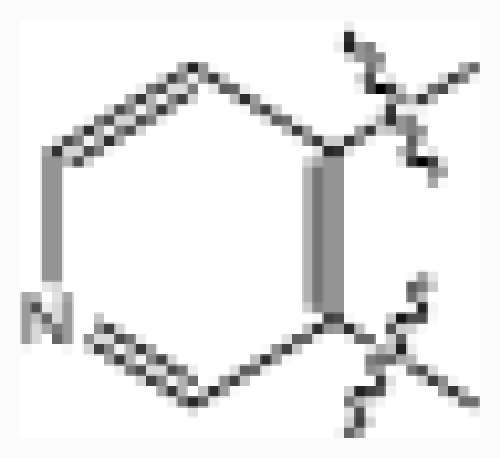						0.006	8.222

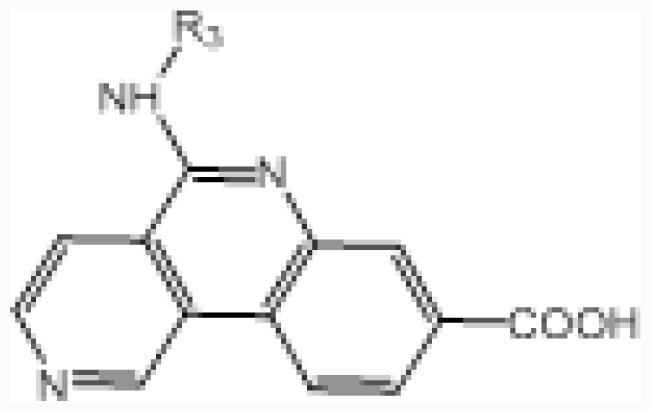								

**No.**	**R_3_**						**IC_50_(μM)**	**pIC_50_**

26	–(CH_2_)_2_NMe						0.025	7.602
27 †	–cyclopentyl						0.027	7.569
28	–OMe						0.008	8.097
29	–cyclopropyl						0.016	7.796
30	–(CH_2_)_2_O-i-Pr						0.011	7.959
31	–(CH_2_)phenyl						0.009	8.046
32 †	–(CH_2_)_2_phenyl						0.003	8.523
33	–(CH_2_)_3_phenyl						0.016	7.796
34	–(3-MeO-phenyl)						0.004	8.398
35	–(3-Cl,4-F-phenyl)						0.004	8.398
36	–(3-F-phenyl)						0.005	8.301
37 †	–(2-Cl-phenyl)						0.008	8.097
38	–(3-Cl-phenyl)						0.001	9.000
39	–(4-Cl-phenyl)						0.007	8.155
40	–(3-acetylenyl-phenyl)						0.003	8.523
41	–(3-CN-phenyl)						0.004	8.398
42	–(4-(PhO)-phenyl)						0.069	7.161
43 †	–(3-(PhO)-phenyl)						0.019	7.721
44	–(3-(SO_2_NH_2_)-phenyl)						0.043	7.367

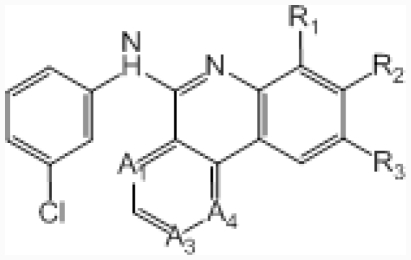								

**No.**	**A_1_**	**A_3_**	**A_4_**	**R_1_**	**R_2_**	**R_3_**	**IC_50_(μM)**	**pIC_50_**

45	–CH=	–N=	–CH=	–H	–C-(1*H*-tetrazol-3-yl)	–H	0.045	7.347
46 ‡	–CH=	–N=	–CH=	–H	–CONH_2_	–H	0.417	6.380
47	–CH=	–N=	–CH=	–Me	–COOH	–H	0.006	8.222
48	–CH=	–N=	–CH=	–H	–H	–COOH	0.350	6.456
49	–N=	–CH=	–N=	–H	–COOH	–H	0.220	6.658
50 †	–N=	–N=	–CH=	–H	–COOH	–H	0.007	8.155

† Test set molecule;

‡ Outlier.

**Table 2:** Summary of Comparative Molecular Field Analysis (CoMFA) and Comparative Molecular Similarity Index Analysis (CoMSIA) results of ligand- and docking-based models about CX-4945 derivatives.

Parameters	Ligand-based alignment		Docking-based alignment	
	
	CoMFA	CoMSIA	CoMFA	CoMSIA
*R*_cv_^2^	0.618	0.681	0.562	0.545
ONC	3	3	4	5
SEP	0.567	0.518	0.616	0.637
*R*_ncv_^2^	0.860	0.828	0.877	0.791
SEE	0.343	0.380	0.326	0.187
*F* value	71.892	56.197	62.523	173.161
*R*_pred_^2^	0.892	0.843	0.556	0.580
Field contribution				
Steric	0.423	0.180	0.668	0.151
Electrostatic	0.567	0.820	0.332	0.246
Hydrophobic	-	-	-	0.321
Acceptor	-	-	-	0.282

*R*_cv_^2^ = Cross-validated correlation coefficient using leave-one-out procedure; *R*_ncv_^2^ = Non-cross-validated correlation coefficient; SEE = Standard error of estimate; *F* = Ratio of *R*_ncv_^2^ explained to unexplained = *R*_ncv_^2^/(1 − *R*_ncv_^2^); *R*_pred_^2^ = Predicted correlation coefficient for the test set of compounds; SEP = Standard error of prediction; ONC = Optimal number of principal components.
